# Melatonin Attenuates LPS-Induced Acute Depressive-Like Behaviors and Microglial NLRP3 Inflammasome Activation Through the SIRT1/Nrf2 Pathway

**DOI:** 10.3389/fimmu.2019.01511

**Published:** 2019-07-02

**Authors:** Burak I. Arioz, Bora Tastan, Emre Tarakcioglu, Kemal Ugur Tufekci, Melis Olcum, Nevin Ersoy, Alper Bagriyanik, Kursad Genc, Sermin Genc

**Affiliations:** ^1^Izmir Biomedicine Genome Center, Izmir, Turkey; ^2^Department of Histology and Embryology, Faculty of Medicine, Dokuz Eylul University, Izmir, Turkey; ^3^Department of Neuroscience, Health Sciences Institute, Dokuz Eylul University, Izmir, Turkey

**Keywords:** melatonin, NLRP3 inflammasome, microglia, lipopolysaccharide, depressive-like behaviors, Nrf2, SIRT1

## Abstract

Inflammation is a crucial component of various stress-induced responses that contributes to the pathogenesis of major depressive disorder (MDD). Depressive-like behavior (DLB) is characterized by decreased mobility and depressive behavior that occurs in systemic infection induced by Lipopolysaccharide (LPS) in experimental animals and is considered as a model of exacerbation of MDD. We assessed the effects of melatonin on behavioral changes and inflammatory cytokine expression in hippocampus of mice in LPS-induced DLB, as well as its effects on NLR Family Pyrin Domain Containing 3 (NLRP3) inflammasome activation, oxidative stress and pyroptotic cell death in murine microglia *in vitro*. Intraperitoneal 5 mg/kg dose of LPS was used to mimic depressive-like behaviors and melatonin was given at a dose of 500 mg/kg for 4 times with 6 h intervals, starting at 2 h before LPS administration. Behavioral assessment was carried out at 24 h post-LPS injection by tail suspension and forced swimming tests. Additionally, hippocampal cytokine and NLRP3 protein levels were estimated. Melatonin increased mobility time of LPS-induced DLB mice and suppressed NLRP3 expression and interleukin-1β (IL-1β) cleavage in the hippocampus. Immunofluorescence staining of hippocampal tissue showed that NLRP3 is mainly expressed in ionized calcium-binding adapter molecule 1 (Iba1) -positive microglia. Our results show that melatonin prevents LPS and Adenosine triphosphate (ATP) induced NLRP3 inflammasome activation in murine microglia *in vitro*, evidenced by inhibition of NLRP3 expression, Apoptosis-associated speck-like protein containing a CARD (ASC) speck formation, caspase-1 cleavage and interleukin-1β (IL-1β) maturation and secretion. Additionally, melatonin inhibits pyroptosis, production of mitochondrial and cytosolic reactive oxygen species (ROS) and nuclear factor kappa-light-chain-enhancer of activated B cells (NF-κB) signaling. The beneficial effects of melatonin on NLRP3 inflammasome activation were associated with nuclear factor erythroid 2–related factor 2 (Nrf2) and Silent information regulator 2 homolog 1 (SIRT1) activation, which were reversed by Nrf2 siRNA and SIRT1 inhibitor treatment.

## Introduction

Major depressive disorder (MDD) is a severe psychiatric disorder that causes considerable socioeconomic burden worldwide (1). According to WHO reports, more than 350 million people globally are affected by depression (2). Complete remission with current anti-depressants occur in only 50% of all cases (3), with frequent serious side effects and requiring long-term therapy for best results. Therefore, there is still need for novel, effective and rapidly acting new antidepressants.

Although, MDD is a very complex disease and its pathophysiological mechanisms are not completely understood, different but interconnected processes including dysregulation of neurotransmission and neurotransmitter alterations, inflammation and inflammasome activation, oxidative stress, and mitochondrial dysregulation are implicated (1, 4). MDD is frequently observed with comorbidities such as age, obesity, systemic inflammation, and infection (5).

Melatonin (N-Acetyl-5-methoxytryptamine) is a hormone produced from L-tryptophan mainly in the pineal gland, but also in peripheral tissues including retina, skin, gastrointestinal tract, and immune cells (6, 7). Its most well-known role in the body is regulating the circadian rhythm, via modulating the gene expression levels of clock genes. In addition, melatonin has anti-oxidant, anti-apoptotic, neuroprotective, and immunomodulatory effects (8). Anti-inflammatory effects of melatonin on activated microglia have already been demonstrated with several *in vitro* and *in vivo* experiments (6, 9). Recent research with brain injury models in mice and rats show that this anti-inflammatory effect is at least partially due to suppression of NLR family pyrin domain containing 3 (NLRP3) inflammasome signaling, where melatonin reduces both expression of components of the NLRP3 inflammasome and the levels of proinflammatory cytokines (10, 11). The exact mechanism of action of melatonin's suppressive effects on NLRP3 inflammasome activity, however, remains to be explored.

Systemic inflammation and neuroinflammation are considered important and crucial components of the pathogenesis of neurodegenerative and psychiatric disorders (12). Microglia, the resident macrophages of the central nervous system (CNS) act as the first line of immune defense in the brain. Microglia activated with internal or external stimuli produce and secrete cytokines and other mediators of inflammation (13). Transient neuroinflammation is beneficial to combat bacteria and their products and to clear cell debris released from injured or dead cells. However, long-lasting microglia overactivation and dysregulated neuroinflammation lead to neuronal cell death and exacerbate the neurodegenerative diseases (14). At present, the exact mechanisms of innate immune responses in the CNS are not fully understood.

NLRP3 inflammasome is a multimeric protein complex that triggers innate immune responses. Assembly of the NLRP3 inflammasome complex is activated by numerous pathogen associated molecular patterns (PAMPs) such as diverse microbial ligands and endogenous danger-associated molecular patterns (DAMPs) such as mitochondrial DNA, Adenosine triphosphate (ATP), reactive oxygen species (ROS), and peptide aggregates (15). In response to these stimuli, cytosolic NLRP3 oligomerizes with other proteins of inflammasome complex, the apoptosis-associated speck-like protein containing CARD (ASC) and pro-caspase-1 (16). This in turn leads to cleavage and maturation of potent pro-inflammatory cytokines, IL-1β and interleukin 18 (IL-18) by mature caspase-1 (15). The activation of NLRP3 inflammasome in myeloid cell types requires first signal termed “priming” to induce transcription of IL-1β and NLRP3. For example, Lipopolysaccharide (LPS) as a DAMP, exhibits priming function via Toll-like receptor 4 (TLR4)/nuclear factor kappa-light-chain-enhancer of activated B cells (NF-κB) pathway; then, a second signal such as ATP activates NLRP3 inflammasome (16).

NLRP3 inflammasome activation leads to caspase-1 dependent pyroptotic cell death in immune cells (17). Active caspase-1 cleaves Gasdermin D (GSDMD), which is a membrane pore forming protein. N-terminal region of pro-GSDMD, the product of cleavage oligomerizes and forms membrane pores. Pore forming activity of GSDMD causes cell-swelling, lysis, release of proinflammatory cytokine IL-1β, and IL-18 into extracellular space and extended immune response (16). Secreted cytokines bind to their cognate receptors leading to autocrine and paracrine effects, and amplification of the inflammatory response.

There are limited *in vivo* and clinical studies that implicate contribution of NLRP3 inflammasome activation in the pathogenesis of depression (16, 18). Behavioral and molecular evidences of DLB being inhibited by NLRP3 inhibitor support that inflammasome activation plays role in the disease pathogenesis (19). Endotoxin-induced DLB model also exhibits NLRP3 inflammasome activation, particularly in the hippocampus (20, 21). Natural product salvianolic acid was shown to suppress microglial activation and neuronal cell death in rats with depression (21). Antidepressant drug fluoxetine decreased microglial NLRP3 inflammation in prefrontal cortex of chronic unpredictable mild stress (CUMS) rats (22). Levels of inflammasome component proteins caspase-1, NLRP3, ASC, and AIM2 mRNA or protein levels are increased in plasma or the peripheral blood mononuclear cells of patients with MDD, compared to non-depressed healthy subjects (23–25). Postmortem evaluation of frontal cortex of patients with bipolar disorders showed higher levels of NLRP3 and ASC in the mitochondrial fractions (26).

ROS is considered as one of the triggers of NLRP3 inflammasome activation (27). ROS are unstable and highly reactive molecules produced by reduction of oxygen mainly during mitochondrial oxidative phosphorylation (27). Excessive ROS production and/or failure of anti-oxidant defense systems result in oxidative stress leading to damage of cellular macromolecules including nucleic acids, proteins, and lipids, and has been implicated in pathogenesis of several diseases (28). Anti-oxidant defense systems consist of several enzymes, some contributing to synthesis and transport of glutathione. Nuclear factor-erythroid 2 (NF-E2)-related factor 2 (Nrf2) is a redox-sensitive transcription factor, that activates the transcription of antioxidant, cytoprotective, and anti-inflammatory genes (29). In normal circumstances, Nrf2 binds to its negative regulator, cytoskeleton-associated protein, Kelch-like ECH-associated protein 1 (Keap1) which ubiquitylates Nrf2 and leads to its degradation (30). In response to oxidative stress, Nrf2 is dissociated from Keap1 and translocates to the nucleus. Heterodimerization of Nrf2 with small Maf proteins in the nucleus activates transcription of its target genes, which contain antioxidant response elements (AREs) in their promoter regions (29). Numerous studies have suggested that Nrf2 activators such as melatonin and natural compounds inhibit NLRP3 inflammasome activation (31, 32). So far, the role of Nrf2 transcription factor in inhibitory effect of melatonin on NLRP3 inflammasome activation in microglial cells remains largely unknown.

Several lines of evidence have shown that Nrf2 deregulation is involved in the pathogenesis of MDD. Two post-mortem studies have demonstrated that Nrf2 levels are decreased in hippocampus, prefrontal cortex, and parietal cortex (33, 34). Consistent with these findings, Nrf2 protein expression were found to be low in the same brain regions of mice with DLB (35). Depression-like phenotype has been observed and cerebral inflammation has been reported in Nrf2 knockout mice (36). And finally, Nrf2 inducers such as sulforaphane, dimethyl fumarate and melatonin blunt these findings (31, 32). However, a mechanistic link between Nrf2-inducing and inflammasome-suppressing actions of these compounds has not been established yet.

It has been reported that melatonin increases SIRT1 expression in the CNS and protects brain in different experimental conditions via activating SIRT1/Nrf2 signaling pathways (37). Silent information regulator 2 homolog 1 (SIRT1) is a well-studied member of Sirtuins, a family of NAD-dependent protein deacetylases regulate a large number of cellular processes including aging, metabolism, redox homeostasis, cell survival, and inflammation (38). SIRT1 deacetylates various target genes including histone proteins, p53, NF-κB, and regulates their activities. It is widely expressed in the CNS and involved in the maintenance of physiological brain functions and exhibits neuroprotective and anti-inflammatory effects in many neurodegenerative diseases. Several studies have shown that SIRT1 deregulation contributes to the pathogenesis of MDD (39, 40). Recent large-scale GWAS studies showed that a SNP close to SIRT1 loci is associated with MDD in Chinese women (41). In addition, SIRT1 mRNA levels were lower in peripheral blood of patients with MDD (42). Pharmacological and genetic inhibition of hippocampal SIRT1 leads depression-like behavior in mice. Injection of SIRT1 inhibitor (sirtinol) and dominant negative SIRT1 expression vector into the DG causes DLB (43). RSV, a SIRT1 activator, attenuates corticosterone, LPS and stress-induced DLB (43–45).

In the present study, our aim was to investigate the effects of melatonin on NLRP3 inflammasome activation *in vivo* and *in vitro* and the role of SIRT1/Nrf2 signaling pathway on anti-inflammatory effect of melatonin. Here, we demonstrated that melatonin decreases sickness behavior and inflammasome activation in LPS-induced DLB mice. Additionally, melatonin inhibits NLRP3 inflammasome activation and pyroptosis in murine microglia via activating SIRT1/Nrf2 signaling pathways.

## Methods

### Chemicals and Reagents

Melatonin and adenosine 5′-triphosphate (ATP) disodium hydrate were obtained from Sigma-Aldrich (St. Louis, USA) and Ultra-Pure LPS (from *E. coli* 0111: B4) was purchased from InvivoGen (San Diego, USA). Sirtinol was purchased from Tocris (Bristol, UK). Fetal bovine serum (FBS), RPMI 1640 cell culture media, L-Glutamine, penicillin/streptomycin, phosphate buffered saline (PBS), trypsin/EDTA were purchased from Biochrom (Berlin, Germany). All the antibodies used in experiments are given in Supplementary Table 1.

### Ethics Statement

All experimental animal studies and animal care were approved by Dokuz Eylül University Izmir International Biomedicine and Genome Institute Local Ethic Committee for Animal Experiments (IBG-AELEC) (Protocol number: 02/2017).

### Animals and Experimental Design

**Figure 1:**
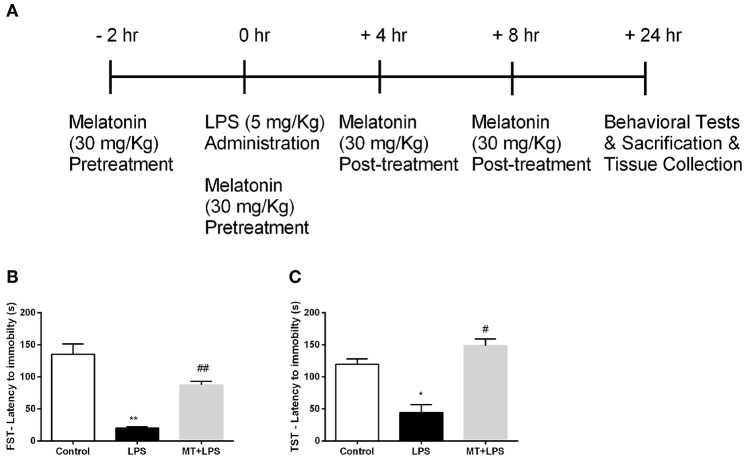
Melatonin treatment ameliorated LPS-induced DLB. **(A)** Schematic representation of experimental design for the LPS-induced inflammasome model. **(B)** Melatonin restored DLB in LPS treated mice in FST, **(C)** Melatonin treated mice also exert less DLB in TST. Data are presented as mean ± S.E.M, *n* = 5. *p < 0.05 and **p < 0.01 compared to control group and ^#^*p* < 0.05 and ^*##*^*p* < 0.01 compared to LPS-induced mice.

Female Balb/c, aged 12–14 weeks, were provided by the IBG-Vivarium. All animals were maintained and housed in the vivarium under controlled conditions (22 ± 2°C; 12 h light/dark periods) with access to food and water *ad libitum*. In the animal experiments, mice were randomly divided into three groups: Control, LPS, Melatonin + LPS. 5 mg/kg doses of LPS were injected intraperitoneally. Melatonin was injected at a dose of 30 mg/kg for 4 times with 6 h-intervals starting 2 h before LPS injection. After 24 h of LPS injection, the experiment was finished and all animals were proceeded to behavioral experiments. After behavioral tests, the mice were sacrificed by decapitation and their brains were collected (Figure 1A). The hippocampus regions of mouse brains were isolated and used for further analysis.

### Behavioral Tests

#### Tail Suspension Test (TST)

The depressive behavior of the mice was analyzed with TST. For the test, the mice were suspended individually by adhesive tape. The mice were isolated from each other. The tape was placed 1 cm from the tip of the tail. The mice were observed for a period of 6 min. Their immobility that is defined as complete motionless was recorded.

#### Forced Swim Test (FST)

Another test, FST, for analysis of depressive behavior of the mice were conducted. For the test, the mice were forced to swim. The experiment was conducted in an open glass cylinder (30 cm height, 10 cm diameter) filled up to 25 cm with water at 25 ± 1°C. The immobility of the mice was recorded for a period of 6 min. The mice were accepted as immobile when they stopped swimming and stayed motionless making only necessary movements for keeping their heads above the water.

### Immunostaining

For the immunofluorescence staining, brain samples were quickly obtained and fixed in 4% PFA solution, then dehydrated in %10 sucrose solution. Later, they were embedded in OCT (Tissue-Tek) and serial sectioned with cryostat. Immunohistochemistry was performed on 10 μm-thick hippocampal sections fixed on slide glass, air dried for 20 min at RT. For permeabilization, slides were fixed with cold acetone for 7 min. Non-specific binding was blocked with 4% donkey serum for 1 h. The slides were washed with 1× TBS containing 0.1% Tween-20 and bound with the primary antibodies incubated at 4°C overnight. Secondary antibodies were incubated for 1 h at room temperature. All the antibodies used are given in Supplementary Table 1. After secondary antibody incubation, tissues were washed 3 times with 1X PBS, then rinsed with dH_2_O. Nuclear staining was performed with Hoechst. Sections were visualized using laser scanning confocal microscopy (LSM880 Confocal Microscope, Zeiss) and fluorescent microscope Olympus BX-61 (Olympus, Japan). Images were analyzed with Image-J software (National Institutes of Health, USA) (46). For intensity measurements, at least 6 regions of interest with equal size for each sample were quantified for each experimental group. Adjustment for standard background across different groups were done. After that, average intensity was calculated and given as arbitrary unit. For co-localization analysis, confocal images taken by Zeiss LSM880 Confocal Microscope with airyscan feature which increase sensitivity, and resolution of the image was used. For quantification of activated microglial cells, whole hippocampal sections in 25x magnified confocal images were analyzed and quantified for Iba1-positive cells. For Iba1-NLRP3 co-localization analysis, JACoP ImageJ plug-in was used (47). To prevent experimental bias, analysis was conducted blind regarding the source of the materials.

### Cell Culture and Treatments

Mouse N9 microglia cells were provided by Dr. Paola Ricciardi-Castagnoli (Toscana Life Sciences Foundation, Siena, Italy) (48). Cells were maintained in RPMI 1640 supplemented with 10% Fetal Bovine Serum (FBS) and 1% L-Glutamine and 1% penicillin-streptomycin at 37°C with 5% CO_2_. N9 microglial cells were pretreated with melatonin for 1 h. Then, treatments with LPS (1 μg/ml) for 4 h and ATP (5 mM) for 1 h were conducted, respectively.

### Lactate Dehydrogenase (LDH) Activity Assay

The release of LDH from N9 microglia cells was determined by Cytotoxicity Detection Kit^PLUS^ (Roche, Germany) by following manufacturer's protocol. Cytotoxicity was determined by using microplate reader Varioskan (Thermo, USA) at 492 nm with a reference wavelength of 630 nm. Cytotoxicity percentages were calculated by the following formula: Cytotoxicity = (OD_Sample_ – OD_LowControl_)/(OD_MaximalRelease_ – OD_LowControl_) ^*^100.

### CCK-8 Cell Viability Assay

Cell viability assay was performed by using Cell Counting Kit-8 (Sigma Aldrich, St. Louis, USA) according to manufacturer's protocol to evaluate the viability of N9 cells. Absorbance values were determined by microplate reader Varioskan Flash (Thermo scientific, USA) at 450 nm with a reference wavelength of 630 nm. Cell viability was given as a percentage absorbance of untreated control cells.

### ELISA

IL-1β and IL-18 levels were determined by enzyme-linked immunosorbent assay (ELISA). IL-1β and IL-18 sandwich ELISA kits (E-Bioscience, USA) were used according to the manufacturer's protocol. The photometric measurements were performed at 450 nm using a microplate reader, Varioskan Flash (Thermo Scientific, USA).

### Quantitative RT-PCR

Total RNA was extracted by using Direct-zol RNA Kit (Zymo Research, USA). Total RNA concentration was measured by a Nanodrop spectrophotometer and 1 μg RNA was used for reverse transcription using cDNA Revert-Aid First Strand cDNA Synthesis Kit (Thermo Scientific, USA) according to the manufacturer's instructions. Quantitative real-time PCR (qPCR) was performed using SYBR-Green I kit and LightCycler® 480 Instrument II (Roche Life Science, USA) according to manufacturer's protocol. The primers used in experiments are given in Supplementary Table 1. Relative expression levels were measured by 2^−ΔΔCt^ formula. Endogenous Glyceraldehyde-3-phosphate dehydrogenase (GAPDH) was used for normalization.

### Protein Extraction

After treatment, total protein from both cell lysate and supernatant were isolated from N9 cells with RIPA lysis buffer (50 mM Tris-HCl, pH 7.4, 150 mM NaCl, 0.25% deoxycholic acid, 1% Nonidet P-40, 1 mM EDTA) including the protease and phosphatase inhibitor (Thermo Scientific, USA). For nuclear and cytosolic fractions, NE-PER Nuclear, and Cytoplasmic Extraction Reagents (Thermo Scientific, USA) were used according to manufacturer instructions. The extracted proteins were stored at −80°C.

### Western Blot Analysis

For western blot analysis, 8–12% SDS–PAGE was used to resolve equal amounts of protein samples from both cell lysate and supernatant. Gel was transferred onto polyvinylidene fluoride (PVDF) membranes (Sigma-Aldrich, USA) and the membranes were blocked with 3% BSA in Tris buffered saline containing 0.05% Tween-20 (TBS-T). Membranes were probed with primary antibodies at 4 °C overnight. Next day, incubated membranes were washed four times with TBS-T, and then incubated with the horseradish peroxidase (HRP)-conjugated secondary antibodies. All the antibodies used are given in Supplementary Table 1. The antigen–antibody complex was screened by chemiluminescence using the Supersignal West Dura ECL reagent (Thermo Scientific, USA). Protein bands were detected with a densitometer (UVP Gel Imager System, CA). Band density analysis was performed with ImageJ software (National Institutes of Health, USA) (46) and normalized to either β-actin and Lamin A/C or given as arbitrary units.

### Caspase-1 Activity Assay

Caspase-1 activity was determined with luminometric Caspase-Glo 1 Inflammasome Assay (Promega, USA) according to manufacturer's protocol. Luminescence was measured with Centro XS^3^ lb 960 microplate luminometer (Berthold Technologies, Germany).

### Propidium Iodide Staining

Microglial cells were seeded into 48 well plate with the density of 3 × 10^4^ cells per well. Following the treatment, the cells were treated with Propidium Iodide (PI) Stain (Thermo Scientific, USA). After 15 min incubation, the PI positive cells were photographed with inverted fluorescent microscope Olympus IX-71 (Olympus, Japan). PI positive cells were counted by ImageJ software and data were expressed as percentage of total cells.

### Immunofluorescence Staining for ASC Specks

Microglial cells were seeded into 25 cm^2^ flasks with the density of 1 × 10^6^ cells per flask. Following the treatment, microglial cells were fixed with 4% paraformaldehyde at 37°C for 15 min, and washed with PBS twice. Permeabilization and blocking were performed with PBS containing 10% FBS and 0.5% Triton-X-100 at 37°C for 30 min. Cells were incubated with ASC primary antibody overnight, followed by Alexafluor-488 conjugated Anti-goat antibody for 1 h. ASC speck images were acquired with LSM 880 Confocal microscopy (Zeiss, Germany). ASC speck positive cells were counted by ImageJ (National Institutes of Health, USA) (46) and data were given as percentage of total cells.

### Detection of Reactive Oxygen Species

Intercellular ROS was measured using CM-H_2_DCFDA (Invitrogen, USA) fluorescent dye. Following the treatments, microglial cells were treated with CM-H_2_DCFDA for 15 min at 37°C. The fluorometric measurements were performed according to manufacturer's protocol using a microplate reader, Varioskan Flash (Thermo Scientific, USA).

### Detection of Mitochondrial ROS

Mitochondrial ROS was detected with MitoSOX (Molecular Probes, Invitrogen, USA), a red fluorogenic dye specifically targeting mitochondria in living cells. For fluorometric assay, treated microglial cells were incubated with MitoSOX (5 μM) for 15 min at 37°C. Absorbance values were measured according to manufacturer's instructions with microplate reader Varioskan (Thermo scientific, Massachusetts, USA). For immunofluorescence assay, treated cells were incubated 5 μM MitoSOX for 15 min at 37°C, and 0.1 μM Hoechst for 2 min for nucleus staining. Images were obtained under inverted fluorescent microscope Olympus IX-71 (Olympus, Japan).

### Analysis of Mitochondrial Membrane Potential

The mitochondrial membrane potential of microglial cells was analyzed using JC-1 stain (Thermo Scientific, USA) according to manufacturer's protocols. JC-1, a cationic stain accumulating on mitochondrial membrane yields red fluorescence in healthy mitochondria whereas it displayed green fluorescence in depolarized or damaged mitochondria. Treated microglial cells were incubated with 2.5 μg/ml JC-1 at 37°C in the dark. FACS Canto II analyzer using a 488 nm laser (Becton Dickinson, USA) was used for assessing the mitochondrial membrane potential. The red/green fluorescence intensity ratio was used to express the change in mitochondrial membrane potential.

### Nrf2 Activation Assay

Nrf2 activity was measured with TransAM Transcription Factor Assay Kit (Active Motif, USA). The experiment was conducted using nuclear extracts according to manufacturer's protocol. The photometric measurements were performed at 450 nm using a microplate reader Varioskan Flash (Thermo Scientific, USA).

### Transfection

The Nrf2 and SIRT1 siRNA were purchased from Dharmacon (Mouse Nrf2 Smartpool ON-TARGET siRNA, L-040766-00-0005 and Mouse SIRT1 Smartpool ON-TARGET siRNA, L-049440-00-0010). The cells were transfected for 48 h using Dharmafect-1 reagent (T-2001-03, Dharmacon, USA) to introduce siRNA according to the manufacturer's protocol.

### Statistical Analysis

Statistical analyses were performed with GraphPad Prism 6.0 (GraphPad Software Inc., CA, USA). Data are presented as mean ± SEM. Comparisons between two groups were done by Mann–Whitney *U*-test. Statistical significance was set at *p* < 0.05. All *in vitro* experiments were performed at least 3 times unless otherwise stated.

## Results

### Melatonin Treatment Ameliorated LPS-Induced DLB

To examine the effects of melatonin on DLB induced by LPS treatment, we performed FST and TST. In FST, while the LPS treatment reduced the latency to immobility, melatonin significantly increased latency to immobility (Figure 1B). The analysis of TST demonstrated that the decrease latency to immobility by LPS treatment were reversed by melatonin treatment (Figure 1C). Overall, these significant changes in behaviors of mice demonstrate that melatonin treatment ameliorates DLB induced by LPS.

### Melatonin Decreased NLRP3 Inflammasome Activation in Mouse Hippocampus

**Figure 2:**
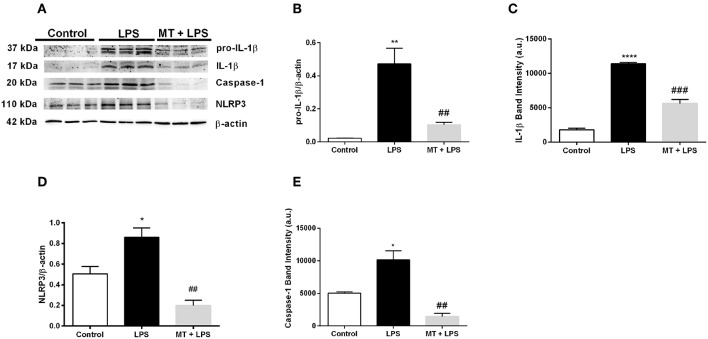
Treatment with melatonin decreased the expression of NLRP3 inflammasome markers in mice hippocampus. **(A–C)** Melatonin treatment decreased both pro-IL-1β and IL-1β levels induced by LPS treatment. **(A,D)** Melatonin suppressed NLRP3 protein levels. **(A,E)** Treatment with melatonin lowered the cleaved caspase-1 levels compared with LPS treated mice. Data are presented as mean ± S.E.M, *n* = 5. *p < 0.05, **p < 0.01 and ****p < 0.0001 compared to control group and ^*##*^*p* < 0.01 and ^*###*^*p* < 0.001 compared to LPS-induced mice.

We next performed Western blotting to analyze NLRP3 inflammasome markers in hippocampus of mice. Melatonin treatment significantly reduced pro-IL-1β and IL-1β protein levels induced by LPS (Figures 2A–C). NLRP3 protein levels also were lower in melatonin treated group compared to the LPS treated group (Figures 2A,D). Lastly, we investigated the effects of melatonin on caspase-1 protein level. Melatonin prevented LPS-induced increase in cleaved form of caspase-1 (p20) level (Figures 2A,E). These results indicate that melatonin treatment inhibits NLRP3 inflammasome activation in the hippocampus of mice.

### Melatonin Ameliorated NLRP3 Inflammasome and Decreased Microglial Activation *in vivo*

**Figure 3:**
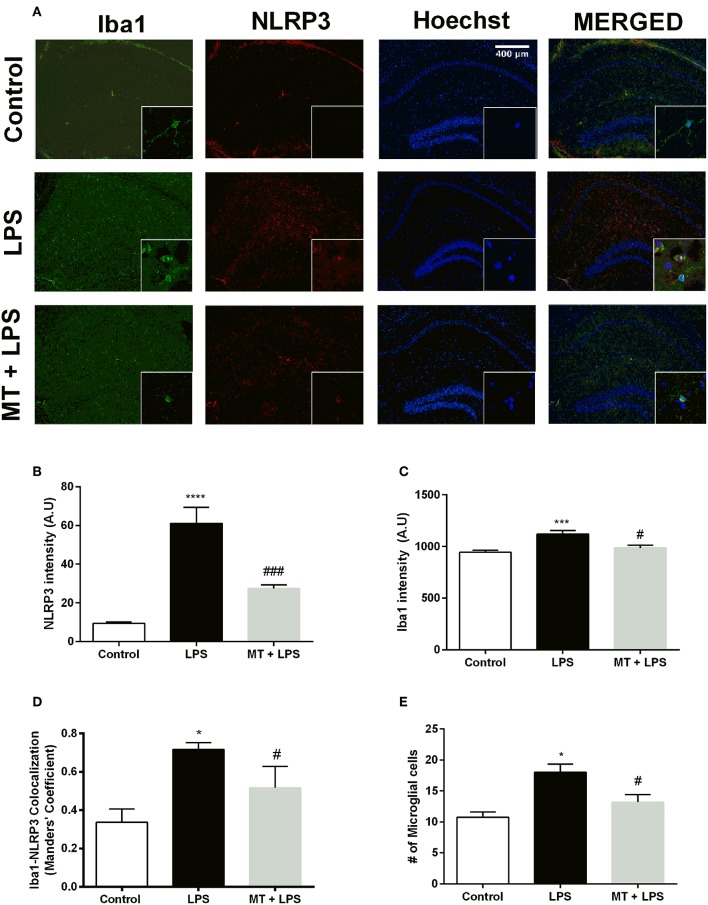
Melatonin treatment inhibited microglial activation and expression of NLRP3. Hippocampal sections were stained with Iba1 as the microglia marker (green) and NLRP3 (red). Nuclei were stained with Hoechst (blue). **(A)** Hippocampal sections were double stained for Iba1 and NLRP3 to localize and assess microglial activation and NLRP3 protein levels. **(B)** Melatonin significantly decreased NLRP3 intensity. **(C)** Increased Iba1 intensity by LPS induction was lowered with melatonin treatment. **(D)** Decreased Iba1-NLRP3 co-localization in melatonin treated mice. **(E)** Number of activated microglia ameliorated by melatonin treatment. Data are presented as mean ± S.E.M, *n* = 5. *p < 0.05, ***p < 0.001 and ****p < 0.0001 compared to control group and ^#^*p* < 0.05 and ^*###*^*p* < 0.001 compared to LPS-induced mice.

To investigate melatonin's effect on NLRP3 inflammasome in hippocampal microglia, immunofluorescence staining for hippocampal sections was performed. Iba1 was used as a specific marker for microglia. As results indicated, LPS treatment dramatically increased NLRP3 expression in the hippocampal region (Figures 3A,B). However, melatonin treatment ameliorated NLRP3 expression compared with that of LPS treated mice (Figures 3A,B). Furthermore, hippocampal microglia were activated by LPS treatment (Figures 3A,C,E). While, Iba1 level in hippocampus was significantly increased in the LPS treated mice, melatonin decreased Iba1 level in the hippocampal region. Although co-localization analysis indicated that all types of cells had NLRP3 expression, it was most abundant in microglial cells (Figure 3D). Additionally, there were significantly fewer Iba1-positive cells in the hippocampal region of melatonin treated mice compared to LPS treated mice (Figure 3E).

We further interrogated melatonin's effect on hippocampal astrocytes. GFAP was used as an astrocyte marker. Results indicated that LPS treatment significantly increased astrocyte activation and accordingly GFAP intensity were increased. However, melatonin treatment inhibited astrocyte activation and decreased GFAP intensity (Supplementary Figures 1A,B).

Lastly we examined the effects of LPS and melatonin on hippocampal neurons via Neurofilament staining. In the LPS treated mice, neurofilament intensity in the hippocampal region was decreased. Melatonin treatment ameliorated this decrease and significantly increased neurofilament intensity (Supplementary Figures 2A,B).

### The Toxicity Curve of Melatonin on N9 Microglial Cells

Non-toxic doses of melatonin were determined with LDH and CCK-8 assays. Melatonin presented no cytotoxicity on N9 microglial cells up to 500 μM. However, a dose-dependent increase was observed in cell death at higher doses (Supplementary Figure 3A). Cell viability also decreased at doses higher than 500 μM of melatonin (Supplementary Figure 3B) in N9 microglial cells. We continued our experiments with 500 μM of melatonin.

### Melatonin Decreased IL-1β and IL-18 on Both mRNA and Protein Levels

**Figure 4:**
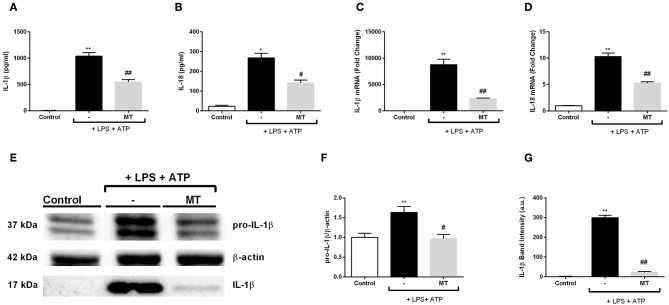
Melatonin reduced mRNA and protein levels of IL-1β and IL-18. N9 microglial cells were pretreated with melatonin (500 μM) for 1 h, then treated with LPS (1 μg) for 4 h and ATP (5 mM) for 1 h. **(A,B)** Suppressor effect of melatonin on secreted IL-1β and IL-18 was measured with ELISA. **(C,D)** mRNA levels of IL-1β and IL-18 reduced by melatonin pretreatment compared with that in LPS and ATP induced cells. **(E–G)** Protein levels of pro-IL-1β and secreted IL-1β were reduced by melatonin compared to LPS and ATP induced cells. Data are presented as mean ± S.E.M, *n* = 5. *p < 0.05, **p < 0.01 compared to control and ^#^*p* < 0.05, ^*##*^*p* < 0.01 compared to LPS and ATP induced cells.

We evaluated the effect of melatonin on secreted NLRP3 inflammasome related cytokines IL-1β and IL-18 with ELISA. Melatonin significantly inhibited IL-1β and IL-18 secretion induced by LPS and ATP (Figures 4A,B). Next, we examined mRNA expression levels of those cytokines. There was a 3.3-fold decrease in IL-1β mRNA level in melatonin pretreated cells compared to LPS and ATP induced cells (Figure 4C). Also, melatonin reduced IL-18 expression level by 2-fold (Figure 4D). Western blot analysis showed that melatonin significantly diminished both pro-IL-1β and mature IL-1β protein levels induced by LPS and ATP treatments (Figures 4E–G).

### Melatonin Diminished NLRP3 Protein Complex Formation by Reducing NLRP3, Caspase-1, and ASC Specks

**Figure 5:**
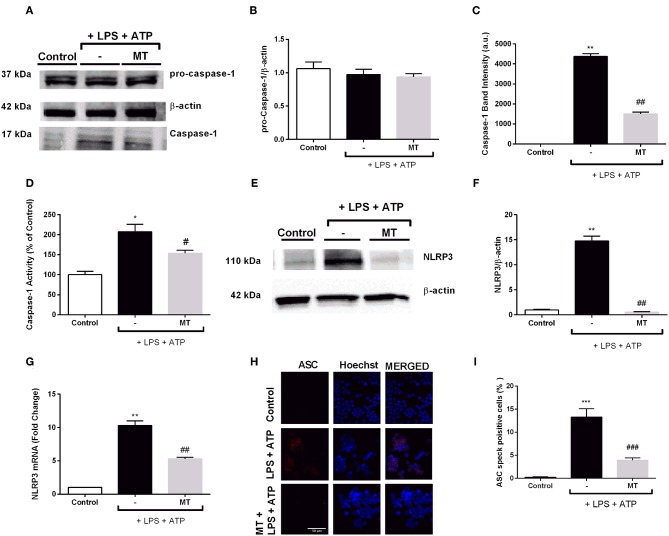
Melatonin reduced NLRP3, caspase-1 and ASC speck formation. N9 microglial cells were pretreated with melatonin (500 μM) for 1 h, then treated with LPS (1 μg) for 4 h and ATP (5 mM) for 1 h. **(A,B)** Pro-caspase-1 show no difference among groups. **(A,C)** Melatonin reduced cleaved caspase-1 in melatonin pretreated cells compared with that in LPS and ATP induced cells. **(D)** Melatonin pretreatment decreased caspase-1 activity compared to LPS and ATP induced cells. **(E–G)** Melatonin suppressed NLRP3 on both protein and mRNA level compared with in that LPS and ATP induced cells. **(H)** ASC speck formation was determined by confocal microscopy. **(I)** Melatonin significantly prevented ASC speck formation compared to LPS and ATP induced cells. Data are presented as mean ± S.E.M, *n* = 5. *p < 0.05, **p < 0.01 and ***p < 0.001 compared to control and ^#^*p* < 0.05, ^*##*^*p* < 0.01 and ^*###*^*p* < 0.001 compared to LPS and ATP induced cells.

Next, we determined the levels of NLRP3 complex proteins to further investigate effects of melatonin on NLRP3 inflammasome in N9 microglial cells. While LPS and ATP treatment increased active caspase-1 (p20) protein levels, melatonin pretreatment significantly inhibited increase in active caspase-1 (p20) (Figures 5A,C). Intracellular level of p45 caspase-1 protein showed no significant difference in all three groups (Figures 5A,B). In addition, we evaluated caspase-1 activity of N9 microglial cells. We found 2-fold increase in caspase-1 enzyme activity with LPS and ATP treatment and melatonin pretreatment significantly decreased caspase-1 activity (Figure 5D). Next, we examined changes in NLRP3 protein and mRNA levels of N9 microglial cells. NLRP3 protein level almost completely diminished in melatonin pretreated cells compared to LPS and ATP induced cells (Figures 5E,F). Besides, melatonin pretreatment reduced NLRP3 mRNA level compared to LPS and ATP induced cells (Figure 5G). ASC speck formation was quantified by immunofluorescence staining. Melatonin ameliorated ASC speck formation which were induced by LPS and ATP (Figures 5H,I).

### Melatonin Protected N9 Microglial Cells Against Pyroptotic Cell Death and Prevented Cleavage of Gasdermin D

**Figure 6:**
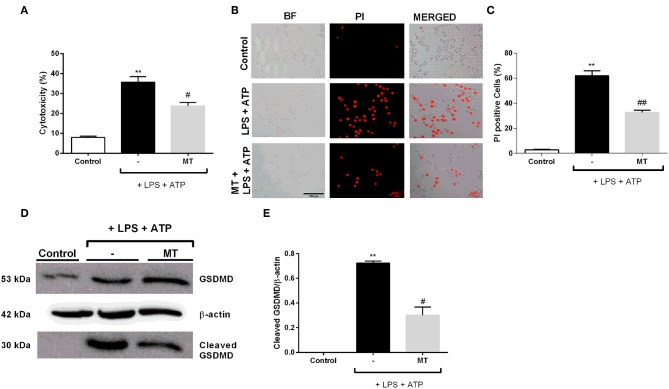
Melatonin prevented pyroptotic cell death. N9 microglial cells were pretreated with melatonin (500 μM) for 1 h, then treated with LPS (1 μg) for 4 h and ATP (5 mM) for 1 h. **(A)** Pretreatment with melatonin inhibited pyroptotic cell death. **(B,C)** Melatonin reduced pyroptotic cell death and decreased PI positive cells. **(D,E)** Melatonin ameliorated GSDMD cleavage induced by inflammasome activation. All the data are presented as mean ± S.E.M, *n* = 5. **p < 0.01 compared to control and ^#^*p* < 0.05 and ^*##*^*p* < 0.01 compared to LPS and ATP induced cells.

We examined the protective effects of melatonin on LPS and ATP induced N9 microglial cells by LDH assay. The treatment with LPS and ATP significantly increased cell death in N9 cells. However, pretreatment with melatonin mitigated LPS and ATP induced cell death (Figure 6A). The pyroptotic cell death was assessed by PI staining. LPS and ATP treatment dramatically elevated pyroptotic cell death. However, melatonin pretreatment prevented NLRP3 inflammasome induced pyroptotic cell death (Figures 6B,C). Next, we examined effects of melatonin on Gasdermin D cleavage by Western blotting. Pretreatment with melatonin significantly reduced cleaved Gasdermin D level compared with that in LPS and ATP induced cells (Figures 6D,E).

### Melatonin Ameliorated ROS Production and Restored Mitochondrial Membrane Potential

**Figure 7:**
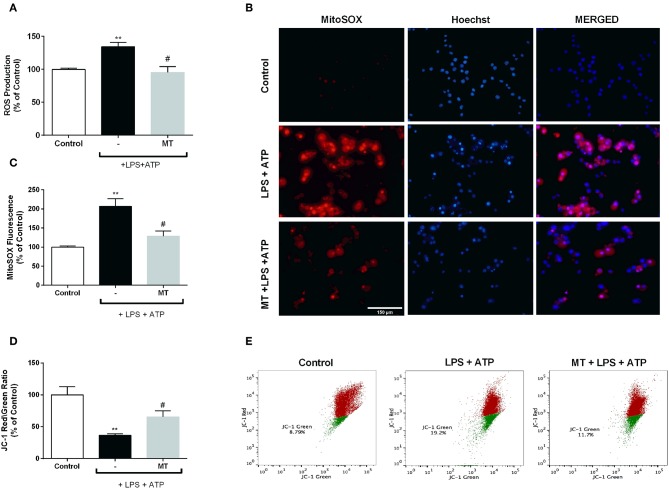
Melatonin inhibited intracellular and mitochondrial ROS production and restored mitochondrial membrane potential. N9 microglial cells were pretreated with melatonin (500 μM) for 1 h, then treated with LPS (1 μg) for 4 h and ATP (5 mM) for 1 h. **(A)** Melatonin pretreatment reduced intracellular ROS production. **(B,C)** Melatonin also decreased mitochondrial ROS production in melatonin pretreated cells compared with LPS and ATP induced cells. **(D,E)** Melatonin pretreatment restored mitochondrial membrane potential. Data are presented as mean ± S.E.M, *n* = 5. **p < 0.01 compared to control and ^#^*p* < 0.05 compared to LPS and ATP induced cells.

To assess the effect of melatonin on intracellular ROS production, we measured ROS level using CM-H_2_DCFDA in N9 microglial cells. While intracellular ROS level significantly increased in LPS and ATP induced cells, pre-treatment with melatonin reduced production of intracellular ROS (Figure 7A). Furthermore, we examined mitochondrial ROS production and mitochondrial membrane potential in N9 microglial cells. Mitochondrial ROS level was increased with LPS and ATP treatment. However, melatonin pretreatment significantly decreased production of mitochondrial ROS to basal level (Figures 7B,C). The mitochondrial membrane potential was monitored with JC-1 staining. Melatonin pretreatment significantly restored membrane potential which was disrupted with LPS and ATP treatment (Figures 7D,E).

### Melatonin Decreases M1 Polarization of Microglia

The effect of melatonin on microglia polarization after LPS and ATP stimulation was investigated *in vitro* by considering that there may be a relationship between its NLRP3 inflammasome inhibitory effect and modulatory effect on microglia polarization. First, we demonstrated that LPS and ATP promoted M1 polarization of N9 microglia and melatonin pretreatment reversed the increase in M1 polarization markers. Furthermore, we checked the M2 state marker CD206 by RT-PCR, and we found that melatonin alone caused polarization into M2 phenotype, but did not alter M2 polarization of microglia in the presence of LPS and ATP (Supplementary Figures 3E–G).

### Melatonin Reduced NF-κB Activation

**Figure 8:**
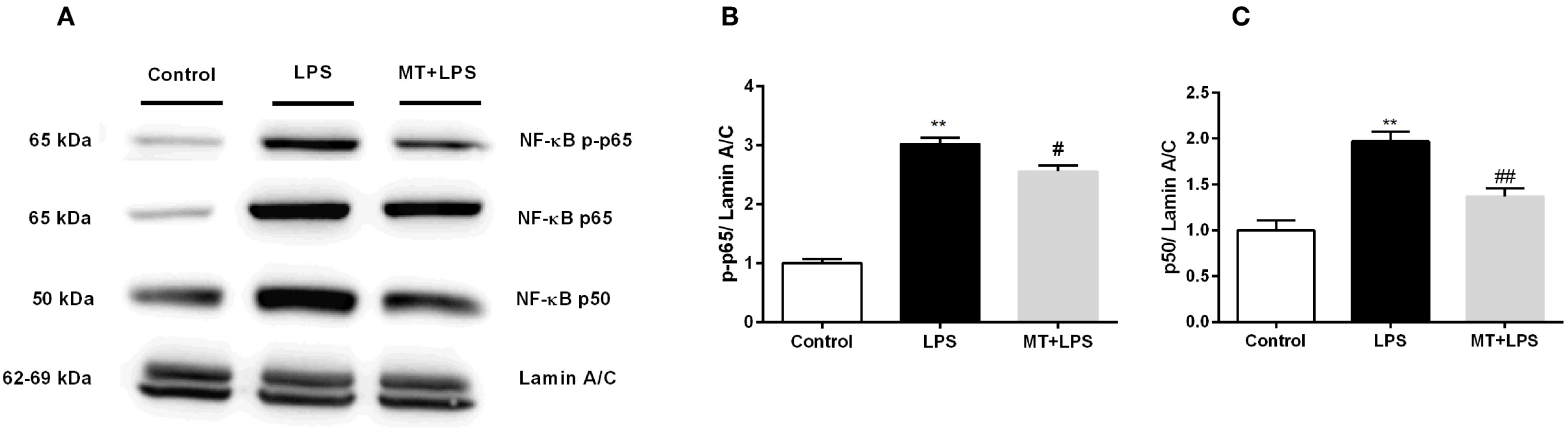
Melatonin inhibited activation of NF-κB. N9 microglial cells were pretreated with melatonin (500 μM) for 1 h, then treated with LPS (1 μg) for 30 min. **(A,B)** Melatonin pretreatment reduced phosphorylation of p65 subunit of NF-κB. **(A,C)** Melatonin pretreatment significantly reduced p50 subunit of NF-κB. Data are presented as mean ± S.E.M, *n* = 5. **p < 0.01 compared to control and ^#^*p* < 0.05 and ^*##*^*p* < 0.01 compared to LPS-induced cells.

To investigate melatonin's effects on NF-κB pathway, we performed Western blotting. While LPS treatment significantly increased p-p65 and p50 subunit levels of NF-κB in nucleus, melatonin treatment inhibited these changes (Figures 8A–C).

### Nrf2 Is Activated by Melatonin and Involved in Protection Against NLRP3 Inflammasome Activation

**Figure 9:**
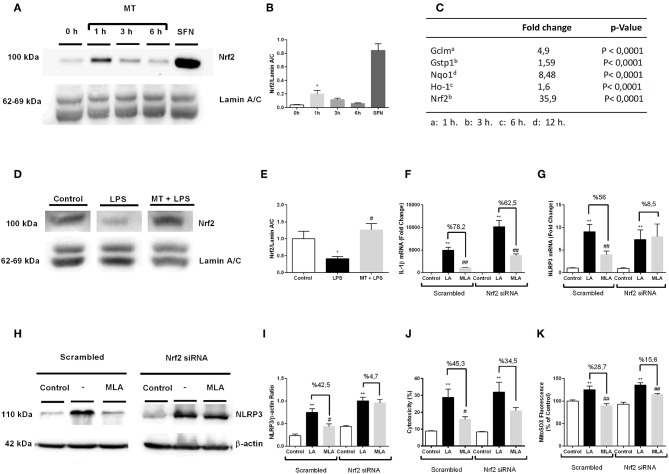
Nrf2 transcription factor is translocated to nucleus by melatonin and involved in protection against NLRP3 inflammasome activation. N9 microglial cells were treated with melatonin (500 μM) for 0–6 h. **(A,B)** Melatonin induced translocation of Nrf2 transcription factor to nucleus. Sulforaphane was used as positive control. **(C)** Nrf2 target genes were upregulated with melatonin treatment. **(D,E)** Melatonin increased translocation of Nrf2 in hippocampus. siRNA-mediated Nrf2 knockdown reversed the protective effect of melatonin against **(F)** Il-1β mRNA level, **(G)** NLRP3 mRNA level, **(H,I)** NLRP3 protein level, **(J)** pyroptotic cell death, **(K)** mitochondrial ROS production. Data are presented as mean ± S.E.M, *n* = 5. *p < 0.05 and **p < 0.01 compared to control, ^#^*p* < 0.05, ^*##*^*p* < 0.01, compared to LPS and ATP induced cells.

To evaluate the possible role of Nrf2 transcription factor on protective effect of melatonin against NLRP3 inflammasome activation, we examined the effects of melatonin on Nrf2 translocations and regulation of Nrf2 target genes. First, we demonstrated melatonin's effect on Nrf2 translocation by Western blotting over the course of a given time frame (Figures 9A,B). One-hour treatment with melatonin significantly increased Nrf2 translocation to nucleus. Next, we performed qPCR to examine the effects of melatonin on expression of Nrf2 target genes including Ho-1, Nqo1, Gstp1, Gclm (Figure 9C). The expression levels of Nrf2 target genes Ho-1, Nqo1, Gstp1, Gclm were significantly upregulated upon melatonin treatment (1.6-, 8.48-, 1.59-, 4.9- fold, respectively). We, further, checked the Nrf2 expression in hippocampus by Western Blotting. While LPS treatment decreased the levels Nrf2, melatonin reversed the effect of LPS and increased Nrf2 levels in hippocampus (Figures 9D,E). After that, we investigated whether siRNA-mediated Nrf2 knockdown would affect the protective effects of melatonin against NLRP3 inflammasome activation (Figures 9F–K). Nrf2 siRNA knockdown efficiency were more than 2-fold (Supplementary Figures 3C,D). Knocking down of Nrf2 increased IL-1β, NLRP3 mRNA levels compared with scrambled groups (Figures 9F,G). We also demonstrated that Nrf2 knockdown completely reversed melatonin's effects on NLRP3 protein levels (Figures 9H,I). Additionally, we examined whether siRNA-mediated knockdown of Nrf2 affects melatonin's protective effect on pyroptotic cell death. Nrf2 knockdown reversed melatonin's effect on pyroptotic cell death (Figure 9J). Lastly, we checked the effect of siRNA-mediated Nrf2 knockdown on mitochondrial ROS production. Nrf2 knockdown diminished the protective ability of melatonin against mitochondrial ROS production (Figure 9K).

### Inhibition of SIRT1 Activation by Sirtinol Reversed Protective Effects by Melatonin Against NLRP3 Inflammasome Activation

**Figure 10:**
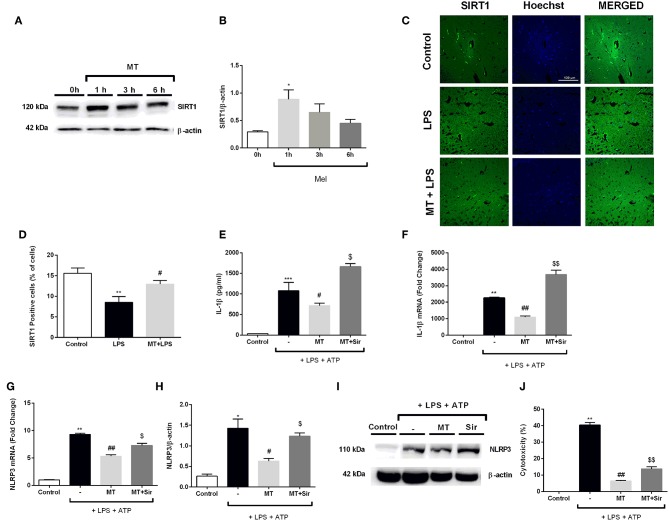
Inhibition of SIRT1 activation by sirtinol reversed melatonin effects on NLRP3 inflammasome activation. N9 microglial cells were treated with melatonin (500 μM) for 0–6h. **(A,B)** Melatonin significantly increased SIRT1 expression. **(C,D)** SIRT1 immunofluorescence staining showed that melatonin increased SIRT1 positive cells in hippocampus **(E,F)** Inhibition of SIRT1 reversed the melatonin's effect on IL-1β mRNA and protein production. **(G)** Sirtinol also increased mRNA levels of NLRP3. **(H,I)** Pretreatment with sirtinol promoted the production of NLRP3 **(J)** melatonin's protective effects against pyroptotic cell death were reversed with inhibition of SIRT1. Data are presented as mean ± S.E.M, *n* = 5. *p < 0.05, **p < 0.01 ***p < 0.001 and compared to control, ^#^*p* < 0.05 and ^*##*^*p* < 0.01 compared to LPS and ATP induced cells and ^*$*^*p* < 0.05 and ^*$$*^*p* < 0.01 compared to melatonin pretreated cells.

In order to evaluate the role of SIRT1 pathway in modulating melatonin's effect on NLRP3 inflammasome activation, we first examined the effects of melatonin on SIRT1 protein expression level over the course of a given time frame. Melatonin significantly increased SIRT1 protein levels in 1 h (Figures 10A,B). We also checked SIRT1 expression in hippocampal region in mice. Melatonin increased SIRT1 positive cells *in vivo* which was decreased by LPS treatment (Figures 10C,D). After that, we checked whether SIRT1 inhibition could affect melatonin's effects on NLRP3 inflammasome activation *in vitro*. Sirtinol reversed the effects of melatonin on LPS and ATP treated cells, increasing protein and mRNA levels of IL-1β (Figures 10E,F). Sirtinol treatment also significantly increased NLRP3 mRNA level, which were reduced by melatonin pretreatment (Figure 10G). Furthermore, Western blot results demonstrated that NLRP3 levels were increased when SIRT1 was inhibited (Figures 10H,I). Then, we examined the involvement of SIRT1 on pyroptotic cell death. Treatment with sirtinol significantly increased pyroptotic cell death by countering the protective effects of melatonin on LPS and ATP treated cells (Figure 10J).

### The Crosstalk Between Nrf2 and SIRT1

**Figure 11:**
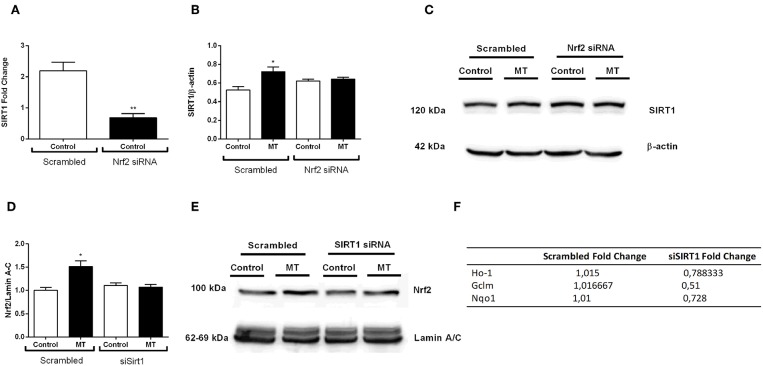
Nrf2 and SIRT1 pathways are co-modulate melatonin effects. N9 microglial cells were treated with melatonin (500 μM) for 1 h. **(A)** siRNA-mediated Nrf2 knockdown significantly decreased SIRT1 expression N9 microglial cells. **(B,C)** Inhibition of Nrf2 reversed the melatonin's effect on SIRT1 protein expression. **(D,E)** siRNA-mediated SIRT1 knockdown significantly decreased Nrf2 translocation. **(F)** Inhibition of SIRT1 significantly downregulated Nrf2 target genes expression. Data are presented as mean ± S.E.M, *n* = 5. *p < 0.05, **p < 0.01 compared to control.

After we investigated melatonin's effects on Nrf2 and SIRT1 pathway, we postulated a possible crosstalk between two protective pathways. Thus, we used siRNA-mediated knockdown of both pathways to demonstrate possible relationship between them. The results indicated that siRNA-mediated Nrf2 knockdown inhibited basal SIRT1 expression in N9 microglial cells (Figure 11A). Additionally, melatonin was not able to increase SIRT1 expression when Nrf2 was knocked down (Figures 11B,C). Next, we conducted siRNA-mediated SIRT1 knockdown in N9 microglial cells. When SIRT1 was knocked-downed, Nrf2 translocation was reduced (Figures 11D,E). Subsequently expression of Nrf2 target genes were downregulated including HO-1, Gclm and Nqo1 (Figure 11F).

## Discussion

In the present study, we showed that melatonin ameliorated LPS-induced behavior abnormalities in a mouse model of acute systemic inflammation and depression, and decreased NLRP3 inflammasome activation in mice hippocampi as evidenced by qPCR, Western blot and immunofluorescence staining. *In vitro* functional experiments confirmed these results and for the first time revealed the beneficial actions of melatonin are partly and significantly dependent on Nrf2 and SIRT1 activation in LPS and ATP challenged murine microglia.

Existing animal models of depression are not ideal since MDD is a very complex disease. However, current animal models based on induction with LPS administration or chronic stress are useful for screening candidate anti-depressive drugs and investigating disease pathogenesis. LPS, widely used to stimulate innate responses, is a lipophilic molecule and may cross to the brain through healthy or destructed blood brain barrier (BBB). Circumventricular organs, with more permeable structures than strict BBB, provide an alternative way for entry of LPS. A single systemic injection of LPS induces DLB characterized with behavioral changes similar to symptoms of acute systemic inflammation or infection triggering exacerbation of depression (49). We observed that melatonin improved behavioral abnormalities (increased latency to immobility) in these animals. Validated and standardized behavioral tests quantitatively confirmed our observations and showed consistency with previous studies. A very recent study has shown that melatonin inhibits acute LPS-induced DLB (50). Another study revealed that NLRP3 inflammasome is involved in DLB (18). However, the effects of melatonin on NLRP3 inflammasome activation induced by LPS has not been studied prior to the present study.

In our study, we proposed that NLRP3 inflammasome activation occurs in hippocampal tissue of LPS challenged mice. Hippocampus is one of the important structures that are implicated in mood regulation. The number of microglia significantly differs between different brain regions both in basal and activated states. Hippocampus contains high number of microglia that are ready to be activated by LPS and other microglial stimulants. In addition, recruitment of peripheral immune cells to the hippocampus is higher after BBB damage. Here, we found microglial activation as shown by Iba1 staining and upregulated expression of NLRP3 in hippocampal microglia. Similar findings have been reported by Pan et al. in CUMS induced depression model (22). Our Western blot analysis demonstrated that NLRP3 and cleaved caspase-1 protein levels are also increased in hippocampal tissue extracts in LPS-treated animals. These findings show that tissue NLRP3 inflammasome activation is associated with DLB symptoms. We also found that both pro- and mature forms of IL-1β increased in LPS-treated animals. All of these changes were significantly prevented by melatonin pretreatment.

NLRP3 inflammasome activation contains two different steps, namely priming stage (signal one) that induce mRNA expression and increase protein levels of NLRP3 inflammasome complex molecules, and induction phase (signal 2) in which NLRP3 inflammasome assembly and activation occur. In most of the myeloid cells including microglia, basal levels of NLRP3 and IL-1β protein are very low. There is not a ready protein pool in cytoplasm to activate NLRP3 inflammasome complex. Thus, priming step is started by LPS binding to TLR4 and consequently TLR4/NF-κB signaling pathway is activated, resulting in the activation of inducible NLRP3 and increase of constitutive expression of pro-IL-1β and pro-IL-18 and pro-GSDMD. The main inhibitory effect of melatonin on NLRP3 inflammasome activation occurs by suppression of these genes' expression at transcriptional level. Consistent with findings of previous studies, we found that melatonin inhibits NF-κB translocation. Therefore, we concluded that melatonin prevents priming step of NLRP3 inflammasome activation. After NLRP3 and IL-1β gene and protein levels reach to a threshold level, DAMPs like ATP released from dead or dying neurons, is sensed by NLRP3 cytosolic sensor and NLRP3 inflammasome complex can be activated. As shown by LDH and PI assays *in vitro*, LPS and ATP-induced cell death was characterized by permeabilization of cell membrane (51). Additionally, caspase-1 mediated cleavage of GSDMD increased with LPS and ATP. Together with the increased secretion of IL-1β and IL-18 levels, these findings support the presence of pyroptotic cell death which is reversed by melatonin. GSDMD-mediated pyroptosis is alleviated with melatonin at transcription level through inhibition of NF-κB activity in adipocytes (52). Here, we demonstrated suppressive effect of melatonin on GSDMD-mediated pyroptosis in microglial cells.

ROS is implicated as one of the main triggers of NLRP3 inflammasome activation. Here, we found both total and mitochondrial ROS amounts are increased after LPS and ATP challenging and reversed by melatonin pretreatment in N9 microglia. Thus, melatonin also inhibits signal 2 in NLRP3 inflammasome activation. Both oxidative stress and inflammation are considered crucial elements of acute and chronic neurodegenerative diseases and each of them promotes and amplifies the other one. For instance, proinflammatory master gene NF-κB is a redox sensitive transcription factor (53). Conversely, NF-κB activates genes of pro-oxidant enzymes such as NADPH oxidases and COX2 (53). Inhibition of NF-κB by melatonin and its direct anti-oxidant effects can break this vicious cycle.

The main source of ROS is oxidative phosphorylation in mitochondria (28). Mitochondrial damage resulting from by diverse factors such as Ca^2+^ influx is considered as putative triggers of NLRP3 inflammasome activation (54). A tight relationship between mitochondria and NLRP3 inflammasome activation was evidenced by recent studies (54). As a marker of mitochondrial damage, we observed that mitochondrial membrane potential was increased by LPS and ATP and reversed by melatonin pretreatment, supporting the proposed mitoprotective property of this compound. As a consequence of mitochondrial damage, oxidized mitochondrial DNA is released to cytosol and triggers NLRP3 inflammasome complex activation as a DAMP (54). Thus, mitoprotective effect of melatonin indirectly inhibits signal 2. Melatonin also regulates mitochondrial dynamics and biogenesis (11, 55).

When microglial cells are activated by environmental factors such as cytokines, DAMPs, and PAMPs, they are polarized into distinct phenotypes. M1 microglia aggravate neuroinflammation by releasing pro- inflammatory cytokines. In contrast, M2 microglia play fundamental role in inflammation resolution by secreting anti-inflammatory cytokines (56). There are only few studies focusing on the relationship between inflammasome activation and polarization in myeloid cells (57, 58). Award et al. showed NLRP3 inflammasome activation in M1 polarized human phagocytes and macrophage, but not in M2 state (57). Another study by using murine RAW264.7 macrophages reported that NLRP3 inflammasome activation induced by LPS results in M1 polarization (58). Similar to this result, we found an association between NLRP3 inflammasome activation and M1 phenotype. Inducing effect of melatonin on macrophage and microglia polarization in favor of the anti-inflammatory phenotype M2 is well known (59). This effect has not been tested in inflammasome condition yet. Here, we showed that LPS and ATP switched M1 polarization and melatonin did not reversed M1 phenotype of N9 microglia. Additionally, melatonin alone increased expression of the M2 marker CD 206. But, it did not alter expression of CD206 in the presence of LPS and ATP. These findings indicate that the anti-inflammatory effects of melatonin may be associated in part with its inhibitory effect of M1 polarization.

Melatonin is a very potent superoxide and hydroxyl radical scavenger (6). As mentioned above, this compound also prevents the production of pro-oxidant enzymes by indirect NF-κB inhibition. Another indirect anti-oxidant effect of melatonin is mediated by Nrf2 transcription factor activation. Proteasome inhibition by melatonin prevents Keap-1 mediated proteosomal degradation of Nrf2 and thereby, increases its cytosolic protein level and translocation to the nucleus (31). Indirect Nrf2 activation by melatonin has been shown in many experimental inflammation and inflammasome studies (9). Nrf2 activates transcription of more than several hundreds of genes encoding anti-oxidant detoxification, iron, lipid and glucose metabolism enzymes (29). Anti-oxidant enzymes mediate synthesis and transport of non-enzymatic anti-oxidant defense molecules, mainly glutathione. Here, we found that melatonin promotes translocation of Nrf2 into nucleus and increases target gene expression. The anti-oxidant, anti-inflammatory, and cytoprotective effects of Nrf2 have been shown in various studies (30). Competition of Nrf2 and NF-κB at DNA binding level is one of the anti-inflammatory mechanisms of Nrf2 (60, 61). An important target of Nrf2, HO-1, also shows anti-inflammatory effect by inhibiting NF-κB (62). In addition to these indirect anti-inflammatory mechanisms, melatonin directly inhibits TLR4 in hypoxic microglia (63).

Numerous studies have suggested that Nrf2 activators such as melatonin and other natural compounds inhibit NLRP3 inflammasome via suppressing ROS production (6, 32). Two recent studies have shown that melatonin inhibits NLRP3 inflammasome activation and oxidative stress in experimental acute brain injury (10, 11). However, direct mechanistic link between Nrf2-inducer and NLRP3-suppressor effect of melatonin has not been reported. Direct anti-oxidant effect of melatonin is well-known. Thus, either direct or Nrf2-mediated effects may be responsible for melatonin's anti-inflammasome action. Here, we found that melatonin translocates Nrf2 into the nucleus and activates the expression of its target genes in N9 microglia. Nrf2 has recently been implicated in several other cellular processes including cytoprotection, cell cycle, inflammation, mitochondrial respiration and biogenesis and iron, lipid and carbohydrate metabolism (29). Melatonin also influence these cellular processes and its anti-inflammatory, cytoprotective, and mitoprotective effects may be through its inhibitory action on NLRP3 inflammasome. To confirm the NLRP3 inflammasome-inhibitory effect of melatonin is through activating Nrf2, Nrf2 was knockdown by siRNA. *In vitro* Nrf2 knockdown partly abolished inhibitory effects of melatonin on NLRP3 inflammasome, suggesting that melatonin exerted these effects via activation of Nrf2.

It is well known that SIRT1 is a key molecule that can mediate many functions of melatonin (9, 37). SIRT1 plays an essential role in inflammatory pathogenesis of DLB (43). Therefore, SIRT1 activation might be a possible therapeutic approach in DLB through NLRP3 inflammasome inhibition. In fact, SIRT1 activation by Resveratrol prevented NLRP3 expression and IL-1β cleavage in hippocampus of mice with sepsis-associated encephalopathy (64). Consistent with previous findings (65), our results confirmed that melatonin increases SIRT1 expression in microglia. To further elucidate the role of SIRT1 activation in inhibitory effect of melatonin on NLRP3 inflammasome, we used sirtinol, as SIRT1 inhibitor *in vitro*. As expected, sirtinol partly reversed anti-inflammatory, anti-oxidant, and cytoprotective effects of melatonin. Our findings supported that the protective effects of melatonin are associated with SIRT1 signaling activation. We propose that SIRT1 activation by melatonin can suppress both priming and activation phase of NLRP3 inflammasome.

In the present study, *in vitro* functional experiments showed the presence of a crosstalk between SIRT1 and Nrf2 pathways. Given the importance of these pathways in the pathogenesis of depression and antidepressant-like action of melatonin (40, 66), these studies should also be confirmed in inflammation induced DLB model. The precise mechanisms of the crosstalk between Nrf2 and SIRT1 in the presence of melatonin in murine microglia are not clear. Although further studies are needed to clarify them, we may suggest several possible mechanisms that were shown in different cells. Similar to our findings, bidirectional crosstalk between SIRT1 and Nrf2 have been reported in human renal proximal tubular and glomerular mesangial cells (67, 68). Nuclear localization of Nrf2 is increased by SIRT1 through suppression of p53 in human mesenchymal stem cells (69). SIRT1-activated AMPK phosphorylates and activates Nrf2 in peritoneal macrophage cells (70). Leptin induced Nrf2 activates SIRT1 binding to ARE containing region in SIRT1 promoter (71).

There are some limitations in our study. First, we performed *in vivo* experiments in female mice. Sex influences on behavioral and pathological changes have been addressed in LPS-induced DLB model (72). Confirmation of current results is necessary by future studies using male mice. Second, systemic inflammation was not evaluated in the present study. Although, abnormality of inflammatory parameters in peripheral samples does not necessarily reflect brain inflammation, pathological inflammatory parameters in serum and PBMC have been reported in clinical studies and animal model of depression (73). Third, pathological changes were evaluated in hippocampus region in this study. However, other brain regions especially prefrontal cortex are also affected in depression (74). In further studies, effect of melatonin should also be evaluated at different doses and route of administration. It should be careful about species differences during translation of *in vitro* and *in vivo* findings to human studies. Because, there are critical differences in function, phenotype and gene expression between human and rodent microglia (75).

In conclusion, this study demonstrated that melatonin treatment ameliorates LPS-induced DLB and inhibits NLRP3 inflammasome activation in hippocampus. Melatonin also diminishes microglial inflammasome activation, pyroptotic cell death, and ROS production induced by LPS and ATP. We concluded that Nrf2 and SIRT1 signaling pathways are important signaling cascades involved in preventative and treating effects of melatonin on NLRP3 inflammasome activation in microglia. Although further studies are required to understand effects completely, presented results shed new lights on mechanisms of anti-inflammatory effect of melatonin in NLRP3 inflammasome activation associated with acute systemic inflammation model of depression.

## Data Availability

All datasets generated for this study are included in the manuscript and/or the Supplementary Files.

## Ethics Statement

This study was carried out in accordance with the recommendations of Dokuz Eylül University Izmir International Biomedicine and Genome Institute Local Ethics Committee for Animal Experiments (IBG-AELEC). The protocol was approved by the IBG-AELEC.

## Author Contributions

BA, BT, KT, KG, and SG designed the study. BA, BT, ET, KT, MO, and NE designed and performed the experiments. BA, BT, ET, KT, MO, KG, and SG analyzed and interpreted the data. BT, KG, and SG wrote the manuscript. BA, BT, ET, KT, MO, NE, AB, KG, and SG read and approved final manuscript.

### Conflict of Interest Statement

The authors declare that the research was conducted in the absence of any commercial or financial relationships that could be construed as a potential conflict of interest.
