# Supplementary material for: Melatonin Attenuates LPS-Induced Acute Depressive-Like Behaviors and Microglial NLRP3 Inflammasome Activation Through the SIRT1/Nrf2 Pathway
Source: Front Immunol. 2019 Jul 2;10:1511. doi: 10.3389/fimmu.2019.01511 (PMC6615259; doi:10.3389/fimmu.2019.01511)
Supplement: Supplementary file 1 [file Data_Sheet_1.docx]

**Supplementary Figure 1. Melatonin treatment inhibited astrocyte activation.** Hippocampal sections were stained with GFAP as the astrocyte marker (green) and NLRP3 (red). Nuclei were stained with Hoechst (blue). (A) Hippocampal sections were double stained for GFAP and NLRP3 to localize and assess astrocyte activation and NLRP3 protein levels. (B) Melatonin significantly decreased GFAP intensity. Data are presented as mean ± S.E.M, n = 5. ****p<0,0001 compared to control group and ##p < 0.01 compared to LPS-induced mice.
