# Supplementary material for: Melatonin Attenuates LPS-Induced Acute Depressive-Like Behaviors and Microglial NLRP3 Inflammasome Activation Through the SIRT1/Nrf2 Pathway
Source: Front Immunol. 2019 Jul 2;10:1511. doi: 10.3389/fimmu.2019.01511 (PMC6615259; doi:10.3389/fimmu.2019.01511)
Supplement: Supplementary file 3 [file Data_Sheet_3.docx]

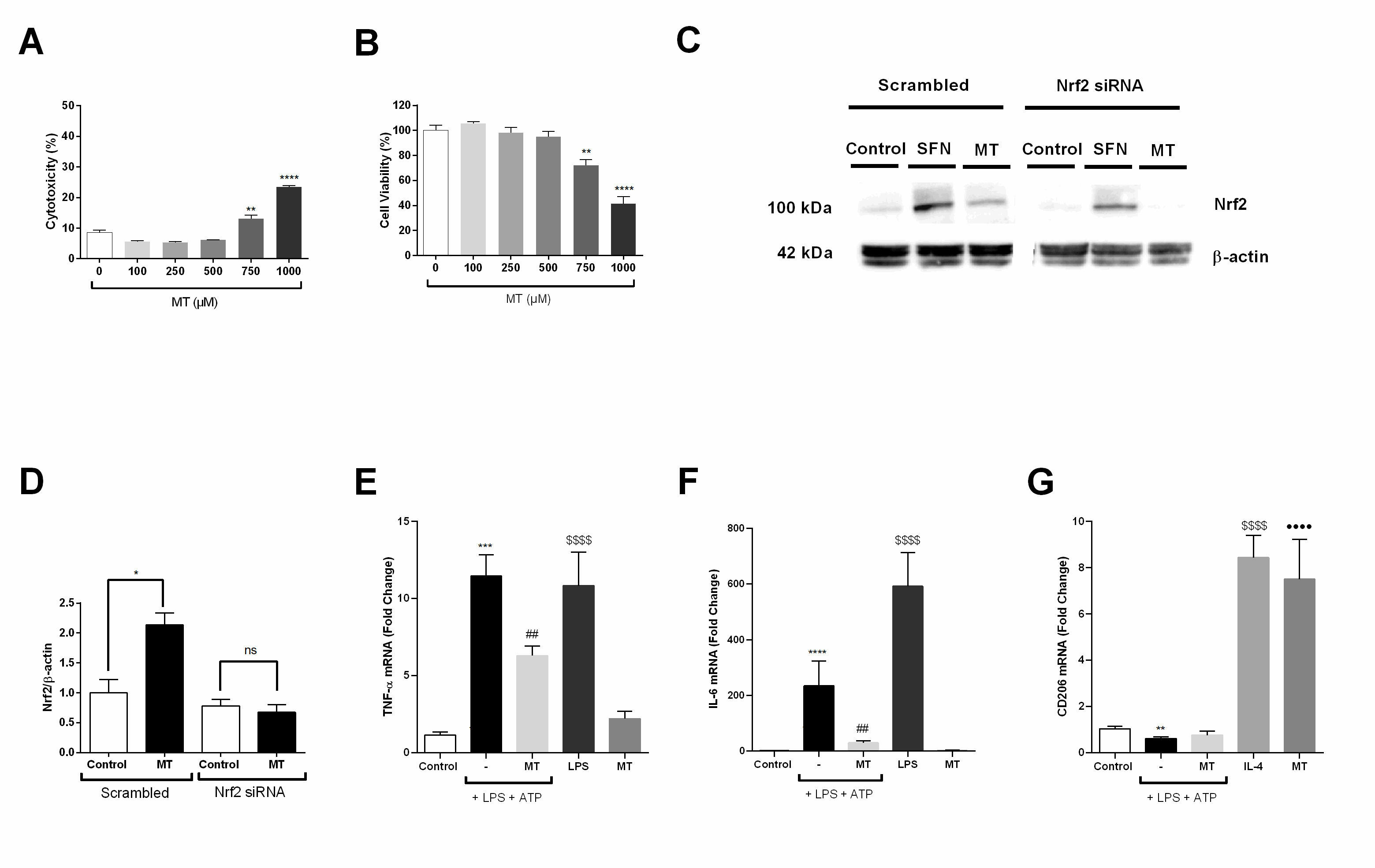


**Supplementary Figure 3. Melatonin’s effect on N9 microglial cells.** Various doses of melatonin were introduced to N9 microglial cells for 6 h. After treatment, **(A)** cell cytotoxicity measured by LDH assay, (**B**) cell viability measured by CCK-8 assay. Higher doses than 500 μM of melatonin were toxic to cells. **(C, D)** Nrf2 transfection efficiency was shown by Western Blotting. **(E, F)** Melatonin treatment reduced M1 polarization state markers Tumor necrosis factor-alpha (TNF-α) and interleukin-6 (IL-6) mRNA levels. **(G)** Melatonin treatment increased M2 polarization state marker CD206. Data are presented as mean ± SEM, n = 5 (*p < 0.05, **p < 0.01. ***p < 0.001, ****p < 0.0001 compared with untreated control; ##p < 0.01 compared with LPS + ATP treatment; $$$$p < 0.0001 compared with untreated control; ••••p < 0.0001 compared with untreated control.
