# Supplementary material for: Melatonin Attenuates LPS-Induced Acute Depressive-Like Behaviors and Microglial NLRP3 Inflammasome Activation Through the SIRT1/Nrf2 Pathway
Source: Front Immunol. 2019 Jul 2;10:1511. doi: 10.3389/fimmu.2019.01511 (PMC6615259; doi:10.3389/fimmu.2019.01511)
Supplement: Supplementary file 4 [file Table_1.docx]

Table 1: Primer and Primary Antibody Lists

| Gene |  | Sequence (5′–3′) | Product Length | Accession |
| --- | --- | --- | --- | --- |
| IL-1β | Forward | TTCTTTTCCTTCATCTTTGAAGAAG | 365 | NM_008361.4 |
|  | Reverse | TCCATCTTCTTCTTTGGGTATTGTT |  |  |
| IL-18 | Forward | CTTTGGAAGCCTGCTATAATCC | 363 | NM_008360.2 |
|  | Reverse | GGTCAAGAGGAAGTGATTTGGA |  |  |
| NLRP3 | Forward | TGCCTGTTCTTCCAGACTGGTGA | 144 | NM_145827.4 |
|  | Reverse | CACAGCACCCTCATGCCCGG |  |  |
| GAPDH | Forward | ACCACAGTCCATGCCATCAC | 452 | NM_001289726.1 |
|  | Reverse | TCCACCACCCTGTTGCTGTA |  |  |
| NQO1 | Forward | TGGCCGAACACAAGAAGC | 262 | NM_008706.5 |
|  | Reverse | TGAATCGGCCAGAGAATGAC |  |  |
| GCLM | Forward | TTGGCTTAGGCATCAGGGTG | 449 | NM_008129.4 |
|  | Reverse | TGTGGTGAGTCCAACTGAGC |  |  |
| GSTP1 | Forward | CGGCAAATATGTCACCCTCA | 301 | NM_013541.1 |
|  | Reverse | GTTCACATGTTCCGGGGAGG |  |  |
| HO-1 | Forward | CACCCTGAGCTGCTGGTGGC | 523 | NM_010442.2 |
|  | Reverse | CAGCCCCTGGGGGCCAGTAT |  |  |
| NRF2 | Forward | TGGACGGGACTATTGAAGGCTG | 735 | NM_010902.4 |
|  | Reverse | GCCGCCTTTTCAGTAGATGGAGG |  |  |
| IL-6 | Forward | GTGGTATCCTCTGTGAAGTCT | 101 | NM_001314054.1 |
|  | Reverse | AAGAGCTTCCAGCCAGTTGCC |  |  |
| TNF | Forward | TCACAGACGAATGACTCCAA | 227 | NM_001278601.1 |
|  | Reverse | GTGCCACTTCATACCAGGAGAA |  |  |
| CD206 | Forward | TGTGGTGAGCTGAAAGGTGA | 118 | NM_008625.2 |
|  | Reverse | CAGGTGTGGGCTCAGGTAGT |  |  |

| Antibody | Company | Catalogue Number | Application | Dilution |
| --- | --- | --- | --- | --- |
| Anti-IL-1β | Abcam | Ab9722 | WB | 1:1000 |
| Anti-Caspase-1 | Abcam | Ab1872 | WB | 1:1000 |
| Anti-NLRP3 | Novus | NBP1-77080 | WB | 1:1000 |
| Anti-NLRP3 | Adipogen | AG-20B-0014 | IF | 1:50 |
| Anti-GSDMD | Abcam | Ab209845 | WB | 1:1000 |
| Anti-NF-κB p65 | Santa Cruz | sc-372 | WB | 1:1000 |
| Anti-NF-κB p-p65 | Cell Signaling | Cst-3033 | WB | 1:1000 |
| Anti-NF-κB p50 | Cell Signaling | Cst-13586 | WB | 1:1000 |
| Anti-IκB | Cell Signaling | Cst-9242 | WB | 1:1000 |
| Anti-Nrf2 | Cell Signaling | Cst-12721 | WB | 1:1000 |
| Anti-SIRT1 | Cell Signaling | Cst-8469 | WB | 1:1000 |
| Anti-Iba1 | Wako | 019-19741 | IF | 1:100 |
| Anti-Neurofilament | Cell Signaling | Cst-2837 | IF | 1:100 |
| Anti-GFAP | Cell Signaling | Cst-12389 | IF | 1:200 |
| Anti-ASC | Santa Cruz | sc-33958 | IF | 1:100 |
| Anti-β-actin | Abcam | Ab8227 | WB | 1:1000 |
| Anti-Lamin A/C | Santa Cruz | sc-20681 | WB | 1:1000 |
| Anti-Rabbit | Abcam | Ab1500073 | IF | 1:500 |
| Anti-Mouse | Abcam | Ab150107 | IF | 1:500 |
| Anti-Rabbit | Cell Signaling | Cst-7074 | WB | 1:2000 |
| Anti-Mouse | Cell Signaling | Cst-7076 | WB | 1:2000 |
